# Menstrual Cycle and Sport Injuries: A Systematic Review

**DOI:** 10.3390/ijerph20043264

**Published:** 2023-02-13

**Authors:** Núria Martínez-Fortuny, Alejandra Alonso-Calvete, Iria Da Cuña-Carrera, Rocío Abalo-Núñez

**Affiliations:** 1 Facultade de Fisioterapia, Universidade de Vigo, 36005 Pontevedra, Spain; 2 REMOSS Research Group, Universidade de Vigo, 36005 Pontevedra, Spain; 3 Fisioterapia Clínica (FS1) Research Group, Instituto de Investigación Sanitaria Galicia Sur (IIS Galicia Sur), SERGAS-UVIGO, 36005 Pontevedra, Spain

**Keywords:** menstrual cycle, sports injuries, sports, athletes

## Abstract

The presence of female athletes has only increased in recent years, as has the incidence of injuries in female sports activities. These injuries are conditioned by multiple factors, including hormonal agents. It is estimated that the menstrual cycle may be related to the predisposition to suffer an injury. However, a causal relationship has not yet been established. The aim of this study was to analyse the relationship between the menstrual cycle and injuries in female sports practice. A systematic search of the scientific literature available in PubMed, Medline, Scopus, Web of Science, and Sport Discus was carried out in January 2022. With 138 articles, only eight studies were found that met the selection criteria for this study. Peak estradiol is associated with increased laxity, strength, and poor use of neuromuscular control. Thus, the ovulatory phase is associated with an increased risk of injury. In conclusion, it seems that hormonal fluctuations throughout the menstrual cycle alter values such as laxity, strength, body temperature, and neuromuscular control, among others. This fact causes women to constantly adapt to hormonal variations, which exposes them to a higher risk of injury.

## 1. Introduction

The female reproductive stage begins at puberty and lasts until the climacteric years. During these years, the menstrual cycle (MC) occurs consecutively [1]. This cycle is a periodic phase that occurs in the female genital organs and is characterised by a series of structural and functional changes induced by hormonal interactions in the hypothalamic-pituitary-ovarian axis [1,2,3,4]. The most characteristic phenomenon of this period is endometrial bleeding, which also allows for measurement of the cycle’s duration [1,2,3,4,5]. It begins on the first day of menstruation and persists until the start of the next period, ranging from 21–35 days [6,7,8]. The duration of uterine bleeding ranges from 4–8 days, and the amount of fluid expelled varies between 30 and 80 mL per cycle [1,2,3,4,5,6,7,8].

The presence of female athletes has only increased in recent years, and the incidence of injury among female athletes has increased accordingly [9]. For example, it is estimated that women are approximately five times more likely to sustain an anterior cruciate ligament (ACL) injury than men [9]. The aetiology of sports injuries is believed to be multifactorial in origin [10], i.e., when these factors align, the risk of injury increases significantly. However, each factor alone is not sufficient to cause injury, but they can cause injury when combined. This model argues that injury is caused by the interaction of environmental, anatomical, biomechanical, and hormonal factors [11]. With regard to environmental factors, it is believed that both the design of shoes and the type of sport can be a conditioning factor for injury [12,13]. For example, the shoulder is among the most injury-prone joints in overhead athletes [14]. Anatomical differences between the sexes may also influence the incidence of sports injuries. A morphological example would be the presence of a more pronounced Q angle in women that, therefore, generates greater overload on the knee ligaments [10]. In terms of biomechanics, there is evidence of neuromuscular control where the quadriceps had greater muscle activation than the hamstrings. This results in anterior slippage of the tibia relative to the femur and may cause injury [15]. However, with the hormonal factors, their influence on sports injuries is still unknown, as there are discrepancies between existing studies that evaluate female hormones and sports injuries [15,16,17,18,19]. Previous research suggests that this higher risk of injuries may be due to the increase in laxity and the effects of the hormones on the tissues, but this is still a hypothesis that should be confirmed in further research [15,16,17,18,19].

For this reason, the present study is focused on analysing the relationship between the hormonal cycle and injuries in sports practice. A secondary objective is to determine at which phase of the cycle women are more susceptible to injury.

## 2. Materials and Methods

In order to carry out this systematic review, an in-depth search was conducted following the guidelines of the “Preferred Reporting Items for Systematic Reviews and Meta-Analyses” (PRISMA) [20] in the following databases: PubMed, Medline, Scopus, Web of Science (WoS), and Sport Discus. The search was carried out in January 2022.

The descriptors used for this search were “menstrual cycle”, “athletic injuries”, “sports injuries”, “wounds and injuries”, “sport”, and “athletes”. All terms are from the Medical Subject Headings (MeSH) thesaurus, except “sports injuries”, which was used as a keyword in Sport Discus. These descriptors were related to each other with the boolean operator “AND”. Table S1 shows the equations used in the different databases mentioned above (Supplementary Material).

In order to select the articles that most closely matched the topic of this systematic review, selection criteria were established. Inclusion criteria were scientific articles in English or Spanish that analysed healthy female athletes. Specifically, only articles published in 2011 or later were included to achieve up-to-date information [21]. Exclusion criteria included repeated studies, studies with a different aim, systematic reviews, case reports, and studies in women with menopause, pregnancy, breastfeeding, or using hormonal contraceptives. Regarding the methodological quality of the studies included, the guidelines of the quantitative study form developed by Law et al. [22] of McMaster University were followed.

## 3. Results

**Figure 1:**
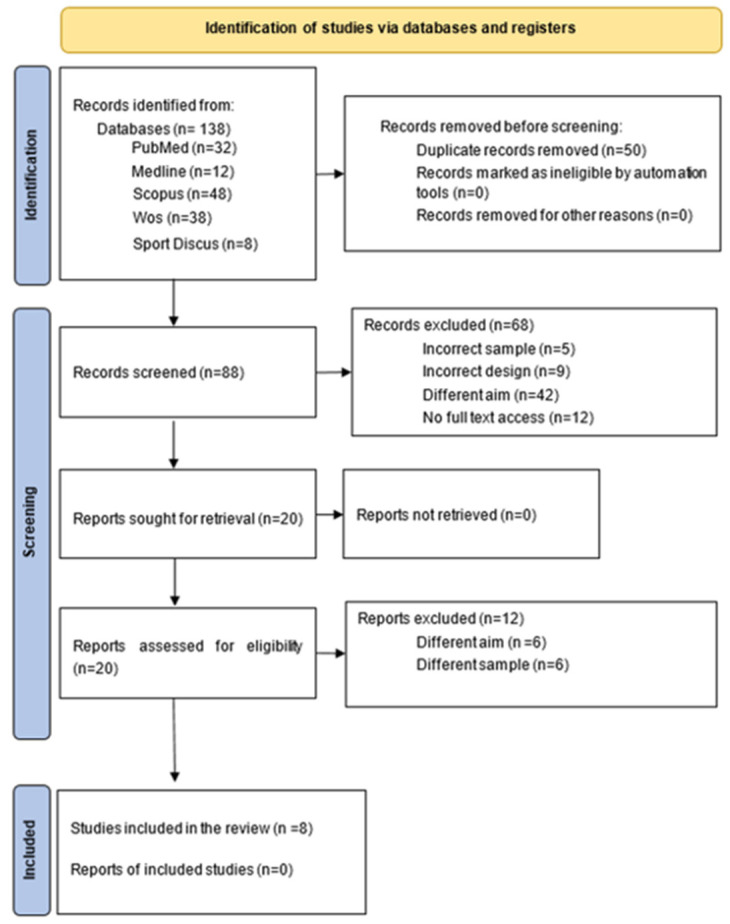
Flow chart (PRISMA).

According to the PRISMA guidelines, the search and selection processes for this systematic review are described in Figure 1, in a flow chart. After the search and the eligibility criteria, eight studies were considered valid and included [23,24,25,26,27,28,29,30]. In addition, the degree of contribution of each study to the systematic review was determined by the quantitative study form described by Law et al. [22], as reflected in Table 1. Thus, the more precise the study is, the more influence it will have on the results.

In this regard, it is shown that the study conducted by Bell et al. [29] was the only one that met all the criteria, making it the reference with the greatest methodological rigour. On the other hand, the remaining studies did not meet all the guidelines established by the form [23,24,26,27,28,30], especially Sommerfield et al. [25], which did not address four fundamental aspects and had the lowest score on the methodological scale.

The relevant data from the articles selected for the systematic review have been compiled and presented in three tables.

Table 2 shows the characteristics of the sample. Detailing this table, the sample size appears to be small in all the articles [24,26,27,28,29,30] except in two of them, which work with samples of 103 participants [25] and 179 subjects [23]. Mostly, the sample was studied without using groups [23,24,25,27,29], except in three articles [26,28,30], where a division was made between experimental and control groups [26,28] in men or women and in participants with or without rupture of the anterior cruciate ligament, respectively. Furthermore, in the study by Schmitz and Shultz [30], they compared knee laxity in two groups of subjects. On the other hand, the mean age is homogeneous, ranging from 14 years [25] to 25.6 years [27]. The physiological characteristics of weight, height, and body mass index are also shown. Regarding the selection criteria of the studies, the most common were: having a normal MC [24,26,29,30], no history of pregnancy [24,27,29,30], and no use of hormonal contraceptives [23,24,26,27,29,30].

Accordingly, Table 2 also shows the characteristics of the sporting activity. The table shows that football [23,28,29] and athletics [25,27,28] were the most popular sports in the studies. Similarly, it also shows the level at which the sporting activity is performed, i.e., at a professional level [23,28] or as a recreational activity [24,25,26,28,28,30], and the frequency of this exercise per week.

Finally, Table 3 shows the characteristics of the studies and the results obtained. With regard to the variables studied, most of them analysed injuries and exposure time [23,25], laxity [24,27,28,29,30], the phases of the MC [23,24,25,27,30], and hormone concentrations [26,27,29]. The tools to assess these variables are shown, generally using ovulation kits [24,27,30], venous blood samples [26,27,29], and the KT knee arthrometer [27,29,30]. The study time is shown, which in most of them covers 1 MC [24,26,27,28,29], with the exception of three articles that delimit it to a school year [25], a sports season [23], or 2 consecutive MCs [30]. The number of measurements taken throughout the study is shown, ranging from 2 measurements [27,28,29] to 4 measurements [26,30], although in the studies conducted by Lagos-Fuentes et al. [23] and Sommerfield et al. [25], the measurements were taken once the season was established. The results obtained by each study are also reported.

## 4. Discussion

Once the results of the seven studies [23,24,25,26,27,29,30] have been analysed, a clear trend appears in terms of the time frame within which injuries occur. The phase most susceptible to injury was found to be the ovulatory phase [24,26,27,29,30], and only one study reported higher risk at the follicular phase [23], while two studies reported higher risk in the luteal phase [24,25]. The reason for such discrepancies between the current results is the difference in establishing the phases of the MC. In agreement, studies used self-reported cycle length to estimate the menstrual phase [23,25]. Despite the discordance, five of the articles found greater injury during the ovulation phase, reinforced by the study of Shagawa et al. [31], which aimed to examine variability in joint laxity as a risk factor for ACL injury during the MCs in 15 female university students with regular MCs. This recent study showed that estradiol concentrations and overall joint laxity were higher in the ovulatory phase. 

Secondly, regarding the etiology of injury in athletes, six studies have investigated the origin of the relationship between injuries and MCs [24,26,27,28,29,30]. In all of them, the results related to ligament laxity were analysed. Four of the six articles witnessed an increase in ligamentous laxity in the ovulatory phase [26,27,27,29,30] and the other two witnessed no change during follow-up [24,28]. This may be explained by the measurement method used to assess laxity, which was performed without an arthrometer and used tests such as the anterior and posterior drawer, the groove sign [24], and the Beighton test [28], which are less objective. Thus, despite identifying changes in laxity, these appear not to be related to the body temperature of the MC. It is known that heat increases the elasticity of a tissue [32,33,34], but in this case, the causal association is not established. Conversely, other studies provided opposite arguments to the above theory [35,36], stating that no association has been reported between MCs and a higher risk of knee injuries.

In this sense, estradiol appears not to be the only hormone that may influence ligament laxity. Other studies have found that relaxin alters ligamentous stiffness. Therefore, relaxin is not only a pregnancy-related hormone. In non-pregnant women, relaxin has been detected during the follicular and luteal phases of the MC. The study conducted by Pearson et al. [37] associated a decrease in patellar tendon stiffness with an increase in blood relaxin levels, and Dragoo et al. [36] observed that athletes with anterior cruciate ligament tears have higher serum relaxin concentrations than those without tears.

Continuing with the injury etiology, variations in strength [24], proprioception [24], and neuromuscular control [26,27] were also found. Forouzandeh et al. [24] highlighted an increase in strength during the ovulatory phase. This fact can be explained by the study conducted by Lowe et al. [38], since they stated that estradiol is closely related to muscle strength. Thus, estrogen seemed to improve the intrinsic quality of skeletal muscle by binding myosin to actin, improving muscle contractions.

In contrast, neuromuscular control was inferior in the follicular [27] and ovulatory phases [26]. Prior research supported the notion that the MC can negatively alter neuromuscular control [39,40,41,42]. These findings might be explained in the ovulation phase, where peak estradiol produces increased quadriceps strength, decreased muscle relaxation time, and increased muscle fatigability. On the other hand, other studies affirmed that estrogen or progesterone may influence the central nervous system [43,44] and, thus, have a negative impact on neuromuscular recruitment.

Finally, Forouzandeh et al. [24] found a decrease in shoulder proprioception in the luteal phase. Due to the lack of studies on this topic, these results could not be compared with similar research. Nevertheless, the aforementioned theories could shed light on this variable [43,44], but further investigation is necessary.

Regarding the characteristics of the participants, the mean age of the women was homogeneous, ranging from 14 years [25] to 25 years [27]. This is compatible with the fact that the mean age of onset of menarche in Spain is 12.6 years [7]. The sample size has generally been small in most of the articles [24,26,27,28,29,30], apart from two of them that work with samples of 103 subjects [25] and 179 participants [23]. This fact should be taken into consideration because the larger the sample size, the greater the internal validity of the research [45,46].

In terms of sporting activity, soccer [23,25,28,29] and athletics [25,27,28] stood out as more popular among other sports [24,26,30]. Both are sports with a high risk of injury [47,48,49,50] as well as being the most practiced sports worldwide [51]. Another aspect to highlight in sports activity is found in the study by Khowailed et al. [27], where physical activity is limited to seven hours or 20 km per week. This fact may be related to the fact that athletic women may experience alterations during their MCs when subjected to a high physical load [52].

Regarding the study variables, a great deal of variability was reported. The MC stood out in all the studies where hormone concentrations or the phases of the cycle were examined. Five of the nine studies examined the knee joint through neuromuscular control [27], tibial acceleration [26], and joint laxity [27,28,29,30], while Forouzandeh et al. [24] assessed the shoulder for strength, proprioception, joint laxity, and functional stability. The studies conducted by Lago-Fuentes et al. [23] and Sommerfield et al. [25] did not assess a specific body part; they looked at the time of exposure to injury and the injured body part. Comparing these aspects with other studies, most of them have studied the knee joint [19,53,54,55,56].

Regarding follow-up time, most of the studies examined the subjects during a normal MC, i.e., 4 weeks [24,26,27,28,29]. Only two studies have been able to perform a more complete approach, over the course of two consecutive indoor soccer seasons [23] and during a school year [25]. On the other hand, other studies in the scientific literature established a follow-up of eight weeks, i.e., two MCs [43,57,58,59,60,61,62] and concluded the need for a longer follow-up time in order to take into account intrasubject variations in the cycle.

This systematic review presents several limitations. First, this study was limited to a specific profile of eumenorrheic female athletes without a history of pregnancy and without hormonal contraceptives, whereas studies in other athletes might provide different results. Second, the sample size of the studies and the heterogeneity of the studies were limited, causing methodological complexity when analysing the association between the MC and sports injuries. Third, the study time was mostly one complete MC, which could be insufficient to determine a relationship. Fourth, sports injuries have a multifactorial ethology, so several factors in addition to the MC could be influencing the results of the studies included in this systematic review. Finally, due to the heterogeneity of the variables and measurements, no meta-analysis was conducted.

Future research in this vein should consider analysing and measuring hormone levels throughout the MC and reporting the use of hormonal contraceptives, which may play a crucial role in injury rates. Relaxin, estrogen, and progesterone concentrations are regulated [47], so it is important to investigate whether hormonal contraceptives could be a protective factor in relation to sports injuries. It has been revealed by the Spanish Association of Midwives [63] that women who have breastfed their offspring can delay their MC until weaning. Therefore, with this research, the question arises as to whether breastfeeding can be a protective factor against sports injuries.

## 5. Conclusions

In conclusion, hormone variations during MCs appear to be related to an increased risk of injury, especially during ovulation. Moreover, laxity, neuromuscular control, and strength oscillate throughout the MC, with these oscillations peaking in the ovulatory phase and coinciding with an estrogenic peak. For these reasons, women need to constantly adapt to hormonal variations, which exposes them to a higher risk of injury.

The lack of scientific evidence on this topic is remarkable, and further research is needed in order to validate these hypotheses. Likewise, sports injuries have a multifactorial etiology, and future research should consider all aspects of female athletes and sports practices in order to prevent them from being injured.

## Figures and Tables

**Table 1:** Methodological quality of the studies included.

Item	Studies							
	Lago-Fuentes et al. [23]	Forouzandeh et al. [24]	Sommerfield et al. [25]	Hohmann et al. [26]	Khowailed et al. [27]	Stijak et al. [28]	Bell et al. [29]	Schmitz, Shultz. [30]
Is the purpose clearly established?	Yes	Yes	Yes	Yes	Yes	Yes	Yes	Yes
Was relevant background literature reviewed?	Yes	Yes	Yes	Yes	Yes	Yes	Yes	Yes
Study design	PCS	TS	PCS	CS	NR	CCS	CS	DS
Sample size	179 W	15 W	103 W	11 W/6 M	12 W	24 W	20 W	38 W
Is the sample described in detail?	Yes	Yes	No	Yes	Yes	Yes	Yes	Yes
Is the sample size justified?	Yes	Yes	No	Yes	Yes	Yes	Yes	Yes
Are the results confident?	No	Yes	No	Yes	Yes	Yes	Yes	Yes
Are the results valid measurements?	Yes	Yes	Yes	Yes	Yes	Yes	Yes	Yes
Is the intervention described in detail?	Yes	Yes	Yes	Yes	Yes	Yes	Yes	Yes
Contamination was avoided?	NR	NR	NR	Yes	NR	NR	NR	NR
Cointervention was avoided?	NR	NR	NR	NR	NR	NR	NR	NR
Are the results meaningful in terms of statistical significance?	Yes	Yes	Yes	Yes	Yes	Yes	Yes	Yes
Were the analyses appropriate?	Yes	Yes	Yes	Yes	Yes	Yes	Yes	Yes
Was the clinical relevance reported?	Yes	Yes	Yes	Yes	Yes	Yes	Yes	Yes
Were the losts reported?	Yes	No	No	No	No	No	Yes	No
Are the conclusions appropriate considering the methods and results?	Yes	Yes	Yes	Yes	Yes	Yes	Yes	Yes

CCS: case and control study; CG: control group; CS: controlled study; DS: descriptive study; M: men; NR: No response; PCS: prospective cohort study; TS: transversal study; W: women.

**Table 2:** Characteristics of the sample, sports practice, and eligibility criteria.

Authors	Characteristics of the Sample						Characteristics of Sports Practice		
	Sample Size	Group	Age (Years)	Weight (Kg)	Height (cm)	BMI (Kg/m^2^)	Type of Sport	Level of Professionality	Exercise Frequency per Week
Lago-Fuentes et al. [23]	179	-	-	59.9 ± 7.8	164.2 ± 5.6	22.2 ± 2.3	Futsal	professional	5–8 h + 1 competition
Forouzandeh et al. [24]	15	-	23.27 ± 1.66	57.60 ± 6.75	167 ± 0.05	-	Overhead athletics	recreational	3 sessions
Sommerfield et al. [25]	103	-	14.0 ± 0.6	57.4 ± 9.8	162.6 ± 5.8	-	Netball, soccer, hockey, lacrosse, swimming, badminton, athletics, and kayak	recreational	3.4 h training + 1 competition + 2.2 h physical training
Hohman et al. [26]	17	EG = 11 W CG = 6 M	16.3 ± 0.7	60.7 ± 6.3	164 ± 6.2	-	EG = netball CG = Rugby	recreational	-
Khowailed et at. [27]	12	-	25.6 ± 3.7	56.8 ± 8.2	160.2 ± 8.6	22 ± 2.2	Athletics	-	No more than 20 Km or 7 h
Stijak et al. [28]	24	EG = 12 W ACL injury CG = 12 W no ACL injury	EG = 24.2 CG = 24.8	-	-	-	Volleyball, athletics, handball, and soccer	18 professional 6 recreational	CG = 4.2 sessions EG = 4 sessions
Bell et al. [29]	20	-	19.6 ±1.3	66.2 ± 9.1	168.6 ± 5.3	-	Soccer	-	-
Schmitz, Shultz. [30]	38	-	G1: 21.4 ± 2.6 G2: 22.5 ± 3.5	G1: 64.4 ± 9.7 G2: 60.2 ± 7.5	G1: 164 ±0.06 G2: 166 ± 0.06	-	-	recreational	2.5–10 h

ACL: anterior cruciate ligament; BMI: body mass index; CG: control group; EG: experimental group; G1: group 1; G2: group 2; M: men; W: women.

**Table 3:** Variables of studies and results.

Authors	Variables	Tools	Intervention Time	Measurements	Results
Lago-Fuentes et al. [23]	Injuries and exposure time MC phases	Excel and calendar	Two sport seasons in soccer	-	191 injuries (104 in training and 87 in matches) 22% in quadriceps 40.8% distension More risk found during the follicular phase.
Forouzandeh et al. [24]	MC phases Strength Proprioception Laxity Stability	Ovulation kit Lafayette hand-held dynamometer Inclinometer ACL test	Complete MC (four weeks)	3: Menstruation, ovulation, and medium luteal phase	Shoulder strength increased significantly during ovulation. Proprioception decreased significantly during the luteal phase. No significant changes were reported in stability or laxity.
Sommerfield et al. [25]	Injuries and exposure time MC phases	OSTRC physical training assistance FITrWoman or My Calendar App	1 year	-	595 injuries in 74 participants 51% in the knee 35% in the ankle No significant relationship was found between the phase of the MC and the risk of injury. A slightly greater tendency to be injured was observed in the luteal phase.
Hohmann et al. [26]	LH, FSH, E2, and P4 Tibial acceleration	Blood sample Acceleration during a jump	Complete MC (four weeks)	3: Menstruation, ovulation, follicular, and medium luteal phase	Higher levels of E2 and AKL were found during ovulation. Lower levels of E2 and AKL were found in CG compared to EG. LH and tibial acceleration were similar in both groups.
Khowailed et al. [27]	MC phases E2 and AKL levels Neuromuscular control	Ovulation kit Blood sample Arthrometer KT-2000 Run 6 min Electromyography	Complete MC (four weeks)	2: follicular and ovulation	Higher levels of E2 and AKL were found during ovulation. In the follicular phase, a higher recruitment in quadriceps was found compared to a lower recruitment in hamstrings when running.
Stijak et al. [28]	E2, P4, AKL, and testosterone	Saliva samples Beighton test	Complete MC (four weeks)	2: luteal and ovulation phases	Women with an ACL injury had significantly lower levels of testosterone and higher levels of E2 and P4.
Bell et al. [29]	E2, P4, AKL, and testosterone Cinematic evaluation of knee and hip	Blood sample Arthrometer KT-2000 Electromyography	Complete MC (four weeks)	2: menstruation and ovulation	Levels of E2, AKL, and P4 were significantly higher during ovulation. No significant changes were found in testosterone levels.
Schmitz and Shultz [30]	MC phases AKL levels	Ovulation kit Arthrometer KT-2000	2 complete MCs (8 weeks)	2: menstruation and ovulation (two times)	Women with higher AKL levels during the ovulation phase also had a significantly higher tolerance to changes.

ACL: anterior cruciate ligament; AKL: anterior knee laxity; CG: control group; EG: experimental group; E2: estradiol; LH: luteal hormone; MC: menstrual cycle; OSTRC: “Oslo Sports Trauma Research Center”; P4: Progesterone.

## Data Availability

Not applicable.

## Supplementary Materials

The following supporting information can be downloaded at: https://www.mdpi.com/article/10.3390/ijerph20043264/s1.

## References

[1] Konovalova E. (2013). El ciclo menstrual y el entranmiento deportivo; una mirada al problema. Rev. UDCA Actual. Divulg. Cient..

[2] Bonen A., Keizer H.A. (1984). Athletic Menstrual Cycle Irregularity: Endocrine Response to Exercise and Training. Phys. Sportsmed..

[3] Wojtys E.M., Jannausch M.L., Kreinbrink J.L., Harlow S.D., Sowers M.R. (2015). Athletic activity and hormone concentrations in high school female athletes. J. Athl. Train..

[4] Moore K.L., Persaud T.V.N. (2013). Embriología Clínica.

[5] Godoy R.C., Pérez L.F., Curbelo S.T., Bosch N. (2004). Fisiología reproductiva femenina: Hormonas sexuales y sus ciclos. BioCancer Res. J..

[6] Fisiología de la Reproducción; AEGO: Madrid, España, 2022. https://aego.es/otra-informacion/fisiologia-de-la-reproduccion.

[7] Curell Aguilà N. (2013). Normalidad y alteraciones de la menstruación en adolescentes. Pediatr. Integral.

[8] Rodríguez Jiménez M.J., Curell Aguilá N. (2017). El ciclo menstrual y sus alteraciones. Pediatr. Integral.

[9] Alanís-Blancas L.M., Zamora-Muñoz P., Cruz-Miranda Á. (2012). Ruptura de ligamento cruzado anterior en mujeres deportistas. An. Med..

[10] Huston L.J., Greenfield M.L., Wojtys E.M. (2000). Anterior cruciate ligament injuries in the female athlete. Potential risk factors. Clin. Orthop. Relat. Res..

[11] Yu B., Kirkendall D.T., Garrett W.E.J. (2002). Anterior Cruciate Ligament Injuries in Female Athletes: Anatomy, Physiology, and Motor Control. Sport. Med. Arthrosc. Rev..

[12] Scranton P.E., Whitesel J.P., Powell J.W., Dormer S.G., Heidt R.S., Losse G., Cawley P.W. (1997). A review of selected noncontact anterior cruciate ligament injuries in the National Football League. Foot Ankle Int..

[13] Hewett T.E., Zazulak B.T., Myer G.D. (2007). Effects of the Menstrual Cycle on Anterior Cruciate Ligament Injury Risk: A Systematic Review. Am. J. Sport. Med..

[14] McFarland E.G., Campbell G., McDowell J. (1996). Posterior Shoulder Laxity in Asymptomatic Athletes. Am. J. Sport. Med..

[15] McLean S.G., Neal R.J., Myers P.T., Walters M.R. (1999). Knee joint kinematics during the sidestep cutting maneuver: Potential for injury in women. Med. Sci. Sport. Exerc..

[16] Wojtys E.M., Huston L.J., Lindenfeld T.N., Hewett T.E., Greenfield M.L. (1998). Association between the menstrual cycle and anterior cruciate ligament injuries in female athletes. Am. J. Sport. Med..

[17] Myklebust G., Maehlum S., Holm I., Bahr R. (1998). A prospective cohort study of anterior cruciate ligament injuries in elite Norwegian team handball. Scand. J. Med. Sci. Sport..

[18] Slauterbeck J.R., Fuzie S.F., Smith M.P., Clark R.J., Xu K.T., Starch D.W., Hardy D.M. (2002). The Menstrual Cycle, Sex Hormones, and Anterior Cruciate Ligament Injury. J. Athl. Train..

[19] Lefevre N., Bohu Y., Klouche S., Lecocq J., Herman S. (2013). Anterior cruciate ligament tear during the menstrual cycle in female recreational skiers. Orthop. Traumatol. Surg. Res..

[20] Page M.J., McKenzie J.E., Bossuyt P.M., Boutron I., Hoffmann T.C., Mulrow C.D., Shamseer L., Tetzlaff J.M., Akl E.A., Brennan S.E. (2021). The PRISMA 2020 statement: An updated guideline for reporting systematic reviews. BMJ.

[21] Guirao Goris S.J.A. (2015). Utilidad y tipos de revisión de literatura. ENE Rev. Enferm..

[22] Law M., Stewart D., Pollock N., Letts L., Bosch J., Westmorland M. (1998). Critical Review Form—Quantitative Studies.

[23] Lago-Fuentes C., Padrón-Cabo A., Fernández-Villarino M., Mecías-Calvo M., Muñoz-Pérez I., García-Pinillos F., Rey E. (2021). Follicular phase of menstrual cycle is related to higher tendency to suffer from severe injuries among elite female futsal players. Phys. Ther. Sport..

[24] Forouzandeh Shahraki S., Minoonejad H., Moghadas Tabrizi Y. (2020). Comparison of some intrinsic risk factors of shoulder injury in three phases of menstrual cycle in collegiate female athletes. Phys. Ther. Sport..

[25] Sommerfield L.M., Harrison C.B., Whatman C.S., Maulder P.S. (2020). A prospective study of sport injuries in youth females. Phys. Ther. Sport..

[26] Hohmann E., Bryant A.L., Livingstone E., Reaburn P., Tetsworth K., Imhoff A. (2015). Tibial acceleration profiles during the menstrual cycle in female athletes. Arch. Orthop. Trauma Surg..

[27] Khowailed I.A., Petrofsky J., Lohman E., Daher N., Mohamed O. (2015). 17β-Estradiol Induced Effects on Anterior Cruciate Ligament Laxness and Neuromuscular Activation Patterns in Female Runners. J. Women’s Health.

[28] Stijak L., Kadija M., Djulejić V., Aksić M., Petronijević N., Marković B., Radonjić V., Bumbaširević M., Filipović B. (2015). The influence of sex hormones on anterior cruciate ligament rupture: Female study. Knee Surg. Sport. Traumatol. Arthrosc..

[29] Bell D.R., Blackburn J.T., Hackney A.C., Marshall S.W., Beutler A.I., Padua D.A. (2014). Jump-Landing Biomechanics and Knee-Laxity Change across the Menstrual Cycle in Women with Anterior Cruciate Ligament Reconstruction. J. Athl. Train..

[30] Schmitz R.J., Shultz S.J. (2013). Anterior Knee Stiffness Changes in Laxity “Responders” Versus “Nonresponders” across the Menstruai Cycle. J. Athl. Train..

[31] Shagawa M., Maruyama S., Sekine C., Yokota H., Hirabayashi R., Hirata A., Yokoyama M., Edama M. (2021). Comparison of anterior knee laxity, stiffness, genu recurvatum, and general joint laxity in the late follicular phase and the ovulatory phase of the menstrual cycle. BMC Musculoskelet. Disord..

[32] Lee H., Petrofsky J.S., Daher N., Berk L., Laymon M., Khowailed I.A. (2013). Anterior cruciate ligament elasticity and force for flexion during the menstrual cycle. Med. Sci. Monit..

[33] Liu S.H., Al-Shaikh R., Yang R.-S., Nelson S.D., Soleiman N., Finerman G.A.M., Lane J.M. (1996). Primary immunolocalization of estrogen and progesterone target cells in the human anterior cruciate ligament. J. Orthop. Res..

[34] Yu W.D., Liu S.H., Hatch J.D., Panossian V., Finerman G.A.M. (1999). Effect of Estrogen on Cellular Metabolism of the Human Anterior Cruciate Ligament. Clin. Orthop. Relat. Res..

[35] Shafiei S.E., Peyvandi S., Kariminasab M.H., Azar M.S., Daneshpoor S.M.M., Khalilian A., Aghajantabar Z. (2016). Knee Laxity Variations in the Menstrual Cycle in Female Athletes Referred to the Orthopedic Clinic. Asian J. Sport. Med..

[36] Dragoo J.L., Castillo T.N., Braun H.J., Ridley B.A., Kennedy A.C., Golish S.R. (2011). Prospective Correlation Between Serum Relaxin Concentration and Anterior Cruciate Ligament Tears among Elite Collegiate Female Athletes. Am. J. Sport. Med..

[37] Pearson S.J., Burgess K.E., Onambélé G.L. (2011). Serum relaxin levels affect the in vivo properties of some but not all tendons in normally menstruating young women: Female tendons and hormone levels. Exp. Physiol..

[38] Lowe D.A., Baltgalvis K.A., Greising S.M. (2010). Mechanisms Behind Estrogen’s Beneficial Effect on Muscle Strength in Females. Exerc. Sport Sci. Rev..

[39] Dedrick G.S., Sizer P.S., Merkle J.N., Hounshell T.R., Robert-McComb J.J., Sawyer S.F., Brismee J.-M., James C.R. (2008). Effect of sex hormones on neuromuscular control patterns during landing. J. Electromyogr. Kinesiol..

[40] Bryant A.L., Crossley K.M., Bartold S., Hohmann E., Clark R.A. (2011). Estrogen-induced effects on the neuro-mechanics of hopping in humans. Eur. J. Appl. Physiol..

[41] Alentorn-Geli E., Myer G.D., Silvers H.J., Samitier G., Romero D., Lázaro-Haro C., Cugat R. (2009). Prevention of non-contact anterior cruciate ligament injuries in soccer players. Part 1: Mechanisms of injury and underlying risk factors. Knee Surg. Sport. Traumatol. Arthrosc..

[42] dos Santos Andrade M., Mascarin N.C., Foster R., de Jármy di Bella Z.I., Vancini R.L., Barbosa de Lira C.A. (2017). Is muscular strength balance influenced by menstrual cycle in female soccer players?. J. Sport. Med. Phys. Fit..

[43] Hewett T.E. (2000). Neuromuscular and Hormonal Factors Associated with Knee Injuries in Female Athletes: Strategies for Intervention. Sport. Med..

[44] Bäckström T., Baird D.T., Bancroft J., Bixo M., Hammarbäck S., Sanders D., Smith S., Zetterlund B. (1983). Endocrinological aspects of cyclical mood changes during the menstrual cycle or the premenstrual syndrome. J. Psychosom. Obstet. Gynaecol..

[45] Leguía-Cerna J.A., Ronald Puescas-Sanchez P., Díaz-Vélez C. (2012). Importace of sample size calculation in research. Rev. Cuerpo Méd. HNAA.

[46] Rondón-Tapía M., Reyna-Villasmil E., Mejia-Montilla J., Reyna-Villasmil N., Torres-Cepeda D., Fernández-Ramírez A. (2017). Hormonas sexuales pre y posparto en preecampticas y embarazadas normotensas sanas. Rev. Perú. Ginecol. Obstet..

[47] Sanchez del Moral R., Herrera Carranza J. (2005). Conocimiento de los medicamentos anticonceptivos en una población universitaria. Segumiento Farmacoter..

[48] Zurita-Cruz J.N., Márquez-González H., Miranda-Novales G., Villasis-Keever M.Á. (2018). Estudios experimentales: Diseños de investigación para la evaluación de intervenciones en la clínica. RAM.

[49] Gómez-Tomás C., Rial Rebullido T., Chulvi-Medrano I. (2021). Estrategias de prevención neuromuscular para las lesiones de ligamento cruzado anterior sin contacto en jugadoras de baloncesto. Revisión narrativa. MHSalud.

[50] Jordan M., Aagaard P., Herzog W. (2017). Anterior cruciate ligament injury/reinjury in alpine ski racing: A narrative review. OAJSM.

[51] Kirkendall D.T., Dvorak J. (2010). Effective Injury Prevention in Soccer. Phys. Sportsmed..

[52] O’Brien D. (1989). Efectos del ejercicio en el ciclo menstrual. Arch. Med. Deporte.

[53] Charlton W.P.H., Coslett-Charlton L.M., Ciccotti M.G. (2001). Correlation of Estradiol in Pregnancy and Anterior Cruciate Ligament Laxity. Clin. Orthop. Relat. Res..

[54] Herzberg S.D., Motu’apuaka M.L., Lambert W., Fu R., Brady J., Guise J.M. (2017). The Effect of Menstrual Cycle and Contraceptives on ACL Injuries and Laxity: A Systematic Review and Meta-analysis. Orthop. J. Sport. Med..

[55] Yanguas Leyes J., Til Pérez L., Cortés de Olano C. (2011). Lesión del ligamento cruzado anterior en fútbol femenino. Estudio epidemiológico de tres temporadas. Apunts. Med. l’Esport.

[56] Murcia-Lora J.M., Esparza-Encina M.L. (2011). La ventana de la fertilidad y marcadores biológicos: Revisión y análisis en ciclos ovulatorios normales. Pers. Bioét..

[57] Duane M., Contreras A., Jensen E.T., White A. (2016). The Performance of Fertility Awareness-based Method Apps Marketed to Avoid Pregnancy. J. Am. Junta Fam. Med..

[58] McLean S.G., Oh Y.K., Palmer M.L., Lucey S.M., Lucarelli D.G., Ashton-Miller J.A., Wojtys E.M. (2011). The Relationship between Anterior Tibial Acceleration, Tibial Slope, and ACL Strain during a Simulated Jump Landing Task. J. Bone Jt. Surg..

[59] Makino A., Garces E., Costa Paz M., Aponte Tinao L., Muscolo D.L. (1998). Evaluación artrométrica de rodilla con KT 1000 en pacientes con ruptura del L.C.A. sin y con anestesia. Rev. Argent. Artrosc..

[60] Shultz S.J., Wideman L., Montgomery M.M., Levine B.J. (2011). Some sex hormone profiles are consistent over time in normal menstruating women: Implications for sports injury epidemiology. Br. J. Sport. Med..

[61] Beynnon B.D., Johnson R.J., Braun S., Sargent M., Bernstein I.M., Skelly J.M., Vacek P.M. (2006). The Relationship between Menstrual Cycle Phase and Anterior Cruciate Ligament Injury: A Case-Control Study of Recreational Alpine Skiers. Am. J. Sport. Med..

[62] Park S.K., Stefanyshyn D.J., Ramage B., Hart D.A., Ronsky J.L. (2009). Alterations in Knee Joint Laxity During the Menstrual Cycle in Healthy Women Leads to Increases in Joint Loads during Selected Athletic Movements. Am. J. Sport. Med..

[63] Rodríguez Rozalén M.A. (2021). Guía Los Consejos de tu Matrona. AEM. https://aesmatronas.com/wp-content/uploads/2021/04/GuiaMatronas_21_150RGB-DEFINITIVO-PUBLI1.pdf.

