# Supplementary material for: Menstrual Cycle and Sport Injuries: A Systematic Review
Source: Int J Environ Res Public Health. 2023 Feb 13;20(4):3264. doi: 10.3390/ijerph20043264 (PMC9958828; doi:10.3390/ijerph20043264)
Supplement: Supplementary file 1 [file ijerph-20-03264-s001.zip › ijerph-2206885-supplementary.pdf]

Table S1. Search equations and databases

	EQUATIONS
**PubMed**	("Menstrual Cycle"[Mesh]) AND "Athletic Injuries"[Mesh]
	(("Menstrual Cycle"[Mesh]) AND "Wounds and Injuries"[Mesh]) AND "Sports"[Mesh]
	(("Menstrual Cycle"[Mesh]) AND "Wounds and Injuries"[Mesh]) AND "Athletes"[Mesh]
**Medline**	(MH "Menstrual Cycle") AND (MH "Athletic Injuries")
	(MH "Menstrual Cycle") AND (MH "Wounds and Injuries") AND (MH "Sports")
	(MH "Menstrual Cycle") AND (MH "Wounds and Injuries") AND (MH "Athletes")
**Scopus**	(TITLE-ABS-KEY (menstrual AND cycle) AND TITLE-ABS-KEY (athletic AND injuries))
	(TITLE-ABS-KEY (menstrual AND cycle) AND TITLE-ABS-KEY (wounds AND injuries) AND TITLE-ABS-KEY (sports))
	(TITLE-ABS-KEY (menstrual AND cycle) AND TITLE-ABS-KEY (wounds AND injuries) AND TITLE-ABS-KEY (athletes))
**WoS**	Menstrual cycle (all fields) and wounds and injuries (all fields)
	Menstrual cycle (all fields) and wounds and injuries (all fields) and sport (all fields)
	Menstrual cycle (all fields) and wounds and injuries (all fields) and athletes (all fields)
**Sport Discus**	(DE "MENSTRUAL cycle") AND (DE "SPORTS injuries")
	((DE "MENSTRUAL cycle") AND (DE "WOUNDS & injuries")) AND (DE "SPORTS")
	((DE "MENSTRUAL cycle") AND (DE "WOUNDS & injuries")) AND (DE "ATHLETES")
